# Mr. Earnest's Case of Singultus

**Published:** 1805-12

**Authors:** Robert Earnest

**Affiliations:** House Surgeon to the Sheffield General Infirmary


### *To the Editors of the Medical and Physical Journal.*

Gentlemen,

As my public situation affords me great opportunities for the registering of cases, I have for some time attended to that kind of study, and I have now selected four from the case-book for publication. If, after you have reviewed them, they should meet with your approbation, and be deemed worthy a place in your very useful miscellany, the

Medical and Physical Journal, you will very much oblige me by inserting them in the next Number.

I am, &c.

ROBERT EARNEST,

*Oct.* 7, 1805. House Surgeon to the Sheffield General Infirmary.

---

No. I. *A Case of incessant Singultus, which continued Five Days successively.*

The subject of this case was a man fifty years of age, had been a free liver, and drank hard; he was emaciated, and had (from the colour of his complexion and tunica conjunctiva) the appearance of a person with chronic hepatitis. There existed much languor and debility in the functions of the body, and a loss of appetite. Fluids were his principal support. The hiccup was in a general way accompanied with pain in the stomach.

During the first four days the following medicines were prescribed, but without the desired effect, viz. æther, opium, camphor, musk, magnesia, and other absorbents. On the fifth day he began with the annexed mixture, which relieved him almost immediately, and in the course of that day carried off the complaint altogether.

℞. Mistur. camphorat. unc. iij. tinct. lavend. comp. spt. ammon. comp. aa. dr. iv. M. fiat mistura, cujus cap. cochl. j. ampl. frequentur indies vel pro re nata.

I visited this patient several times in the night-time and I found that even during sleep, the singultus did not cease, whence I name it incessant.

The stomach in this case was judged to be the seat of the complaint, as the patient very frequently complained of much pain and soreness about the scrobiculus cordis; acid eructations, and sometimes of nausea.

Singultus is said to be a spasmodic affection of the stomach, œsophagus, and muscles subservient to deglutition, and diaphragm; but from what has been stated before, I have every reason to believe that the stomach alone in this case was the seat of the complaint.

No. II. *A Case of Fungus Ulcer.*

T. B. a man forty-nine years of age, of the middle stature, his habit apparently cachectic, complexion sallow, and had lost the vision of the left eye by that disease called by Dr. Cullen Caligo Corneæ.

Some

Some time in the year 1803, he was attacked with a continual dull aching pain, not far above the wrist of the right hand, nearly over the inferior extremities of the radius and ulna. In the course of a few months a tumour gradually took place in the pained part.* Ulceration had been produced by various external acrid applications, that the patient had been advised to make use of, which in the end inflamed and excoriated the outer integuments of the tumour, and shortly afterwards it degenerated into a large fungus ulcer, measuring between eleven and twelve inches in circumference, which was extremely irritable, and would bleed freely upon the slightest touch. This ulcer was the cause of continual pain, and sometimes such a profuse hæmorrhage would come on as to demand the aid of styptics and compression. The loss of so much blood from time to time, with so much continual pain, &c. had reduced, the patient's health and strength very materially. He went on in this manner a very considerable time, his health and strength all the while declining very seriously. At length he was recommended to the Infirmary in the last week of June 1804, but there appeared now no remedy likely to prove salutary, but the operation of amputation of the arm below the elbow, as the parts above the ulcer appeared to be in a sound state. The operation was proposed to the patient and he readily consented to it, and accordingly the arm was removed on the Friday, in the first week of August. The stump healed and cicatrized kindly, and in about a month after the operation, the man was discharged cured.

I first injected and afterwards dissected the parts diseased, in order to ascertain what had been the cause of so much hæmorrhage, and the production of so much fungus.† This was very soon confirmed as I found that the interosseal artery, in that branch which serves the extensor digitorum communis, of the third and fourth fingers, near the end of the ulna, to be diseased and ruptured; the

* This was a fungus tumour, which I suppose in many respects resembled the fungus hæmatodes spoken of by Mr. Hey, in his book entitled Practical Observations in Surgery; wherein he has enumerated many cases of this disease. I think there is a great similarity between this case and that of Ann Wood's, which is Case x. in Mr. H's book, but with this difference, the radius in her case was diseased, but in this case the bones were sound, as will be explained above.

† In cutting into the substance of this fungus, it very much resembled a medullary kind of mass of various colours.

cutaneous vein was in a varicous state, the periosteum and bones were perfectly sound.*

No. III. and IV. *were Cases of Chronic Syphilitic Complaints, wherein the Thyroid Cartilage had become deeply ulcerated.*

One of the subjects of the above cases, No. III. was an American sailor, nearly fifty years of age, and had drank spirituous liquors freely; he had had syphilis frequently; and upon inspecting his body after death, two open buboes were discovered, one in each groin, which the patient had never mentioned, although such an enquiry had been made. The other subject, No. IV. was an Englishman, and was forty-two years of age; he had been abroad a good deal as a drummer in the army; also been in the habit of drinking hard; he likewise had been infected with syphilis several times.

The symptoms of these two cases were so analogous to each other, that I have thought it proper to speak of them jointly, and the symptoms were as follows: viz. general emaciation; a small, weak, quick pulse; loss of appetite; breathing attended with great difficulty, pain and anxiety, and at every inspiration there was a loud crowing of the voice, and he was frequently alarmed by a sense of suffocation, accompanied with flushings of the face; cough and expectoration, wandering pains of the extremities; but whenever either complained, it was chiefly of pain in the throat, pointing at the same time to the upper and front part of it. The difficulty in breathing was much increased in the act of swallowing.

The broken down and emaciated constitutions of these two patients, would not admit of pushing a full and regular mercurial course; however, various slight mercurial remedies, along with tonics, opiates, fumigations with the hydrarg. sulphurat. ruber. and a nourishing diet, were administered, but all without any beneficial or lasting effect, as they both went on gradually declining with what I should term tabes syphilitica, which in the end destroyed them.

As

* Quere.—Is it not probable that the fungus hæmatodes (so skilfully described by Mr. Hey) may be produced and fed by the rupture of some artery, but more particularly some medullary artery, being primarily affected by the caries of bones? as I am pretty sure that I have seen several cases of fungus of this description, where the bones have been carious at the same time.

As these patients so particularly complained of pain about the t yroid cartilage and trachea, I was very solicitous of ascertaining the cause; I therefore dissected out the larynx with part of the trachea; I then examined the parts externally, and found all natural in appearance, excepting that the parts in general being in a state of enlargement. I next laid open the larynx and trachea longitudinally, and discovered the internal membrane to be considerably thickened and inflamed, and was covered with a great quantity of viscid mucus; and the thyroid cartilage itself was deeply ulcerated.*

If what has been discovered in these two cases, should at any future time be the means of pointing out more successful or efficacious remedies in similar cases, I shall be sufficiently rewarded for my trouble in recording and publishing them.

* Being now convinced of the cause of so much pain and uneasiness in the throat, I desisted from further inquiry, notwithstanding, in all probability the whole length of the aspera arteria was affected in the way I have mentioned above; and no doubt the lungs would have been found in a state more or less diseased.

